# Molecular characterization of hemotropic mycoplasmas (*Mycoplasma ovis* and ‘*Candidatus* Mycoplasma haemovis’) in sheep and goats in China

**DOI:** 10.1186/s12917-017-1062-z

**Published:** 2017-05-26

**Authors:** Xiaoxing Wang, Yanyan Cui, Yan Zhang, Ke Shi, Yaqun Yan, Fuchun Jian, Longxian Zhang, Rongjun Wang, Changshen Ning

**Affiliations:** College of Animal Science and Veterinary Medicine, Henan Agricultural University, Zhengzhou, 450002 People’s Republic of China

**Keywords:** Hemoplasmas, *Mycoplasma ovis*, ‘*Candidatus* Mycoplasma haemovis’, Small ruminants, 16S rRNA gene

## Abstract

**Background:**

Hemotropic mycoplasmas (hemoplasmas) are emerging zoonotic pathogens with a worldwide distribution that can cause mild to severe hemolytic anemia, icterus, ill-thrift, infertility, and poor weight gain. However, understanding of the molecular epidemiology of hemoplasmas (*Mycoplasma ovis* and ‘*Candidatus* Mycoplasma haemovis’) is limited in sheep and goats, and the hemoplasma strain/species/variant ‘*Candidatus* M. haemovis’ was poorly studied throughout the world and had never been detected in China until now. Thus, the aim of the present study was to determine the molecular prevalence of hemoplasmas, including *M. ovis* and ‘*Candidatus* M. haemovis’ in sheep and goats from seven provinces and one autonomous region of China.

**Methods:**

A total of 1364 blood samples were collected from sheep and goats in seven provinces and one autonomous region of China. All blood samples were tested for hemoplasmas (*M. ovis* and ‘*Candidatus* M. haemovis’) by nested PCR amplification based on 16S rRNA gene. Positive specimens underwent nucleotide sequencing and phylogenetic analysis.

**Results:**

Overall, 610 specimens (44.7%, 610/1364) were shown to be hemoplasmas (*M. ovis* and ‘*Candidatus* M. haemovis’) -positive by nested PCR amplification based on 16S rRNA gene. The prevalence in goats was 44.1% (379/860), and 45.8% (231/504) in sheep, while that in grazing small ruminants was 54.4% (396/728) and 33.6% (214/636) in house feeding small ruminants. Sequencing of the nearly complete 16S rRNA gene was successful for the 103 randomly selected positive specimens from different farms in different sampling sites of China. Among them, analysis of the 16S rRNA gene sequences identified *M. ovis* (*n* = 56) and ‘*Candidatus* M. haemovis’ (*n* = 47). Two (KU983740 and KU983746) of the four novel genotypes obtained in this study were closely related to *M. ovis*, while the other two genotypes (KU983748 and KU983749) had high identity with ‘*Candidatus* M. haemovis’. Remarkably, the genotype (KU983740) of *M. ovis* in sheep and goats in this study fell in a clade with two human hemoplasmas from USA (KF313922 and GU230144) and shared 99.8%–99.9% with them.

**Conclusions:**

In this study, ‘*Candidatus* M. haemovis’ was first detected in Chinese sheep and goats and hemoplasmas (*M. ovis* and ‘*Candidatus* M. haemovis’) are highly prevalent, and widely distributed in China.

**Electronic supplementary material:**

The online version of this article (doi:10.1186/s12917-017-1062-z) contains supplementary material, which is available to authorized users.

## Background

Hemotropic mycoplasmas (hemoplasmas, formerly classified as *Haemobartonella* and *Eperythrozoon* spp.) are uncultivated, small, pleomorphic, wall-less bacteria that parasitize on the surface of animal erythrocytes [1–3]. Hemoplasmas have now been reclassified into the *Mycoplasma* genus based on 16S rRNA gene sequence. These pathogens can cause mild to severe hemolytic anemia, icterus, ill-thrift, infertility, and poor weight gain, but death is rare in infected adults [1, 4–6]. Worldwide, hemoplasmas have been reported to affect livestock [2, 7–10], companion animals [11–17], wildlife [5, 18, 19], and humans [20–23].

*Mycoplasma ovis* (formerly *Eperythrozoon ovis*), which could elicit major health problems and high mortality in lambs, has been identified in sheep and goats [8, 24–26]. In 2009, a novel hemoplasma, a 17 bp long deletion and an overall the 16S RNA gene sequence identity of only 97% compared to *M. ovis*, has been detected in Hungary. Subsequent studies identified this new hemoplasma as ‘*Candidatus* M. haemovis’ [24]. Subsequently, complete genome sequencing conducted by Deshuillers et al. revealed that *M. ovis* strain Michigan contains two copies of 16S rRNA gene, one corresponding to *M. ovis*, and the other to ‘*Candidatus* M. haemovis’ [27]. Therefore, Deshuillers et al. and other researchers can speculate that *M. ovis* and ‘*Candidatus* M. haemovis’ may exist as a single species with two copies of 16S rRNA gene or as two different species [27].

With an increasing number of hemoplasmosis clinical cases, many countries have suffered extensive losses of livestock. However, few epidemiological surveys on hemoplasmosis in sheep and goats have been conducted during the last decade. The prevalence of *M. ovis* was 6.3% (36/573) in small ruminants in North Africa [28], 20% (4/20) in goats in Switzerland [25], 87% (27/31) in captive cervids in Brazil [26] and 26.3% (5/19) in free-living Japanese serows [18]. Surprisingly, a 49-year-old veterinarian from Texas of USA was found to be co-infected by two *M. ovis* variants, *M. ovis* and ‘*Candidatus* M. haemovis’. In China, *M. ovis* was found in 41.0% (151/371) and 16.1% (192/1191) in small ruminants from Hubei province and Chongqing city, respectively [29, 30]. However, understanding of the molecular epidemiology of hemoplasmas (*M. ovis* and ‘*Candidatus* M. haemovis’) is limited in sheep and goats. And ‘*Candidatus* M. haemovis’ was poorly studied throughout the world and had never been detected in China until now. Thus, the aim of the present study was to determine the molecular prevalence of hemoplasmas, including *M. ovis* and ‘*Candidatus* M. haemovis’ in sheep and goats from seven provinces and one autonomous region of China.

## Methods

### Specimen collection

**Fig. 1:**
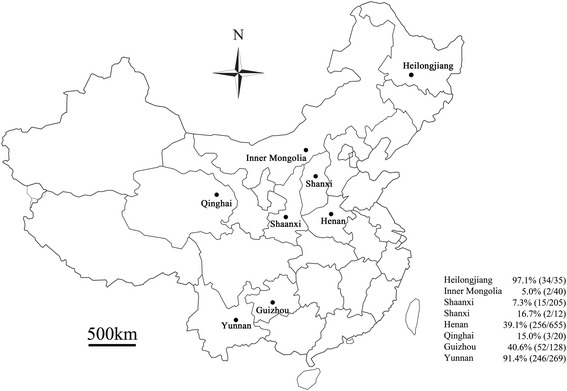
Collection sites (indicated by *black circles*) in China

A total of 1364 EDTA-anticoagulated blood samples were collected from jugular vein of sheep and goats in Henan, Guizhou, Shanxi, Shaanxi, Yunnan, Qinghai, Heilongjiang provinces and the Inner Mongolia autonomous region from March 2012 to May 2015 (Fig. 1 and Additional file 1: Figure S1).

For each animal, one blood sample was collected into an EDTA tube marked with breed, gender, age, and feeding habit of the animal. The animals have no obvious clinical symptoms during blood sampling. The specimens were then transported in an ice box to the laboratory for further processing and analysis.

### DNA extraction

For each specimen, DNA was extracted from 500 μl of anticoagulated whole blood using a Genome DNA Extraction Kit (LifeFeng, Shanghai, China) according to the manufacturer’s instructions. DNA was eluted from the column with 200 μl Tris-EDTA buffer. After DNA extraction, DNA concentration and quality was measured using absorbance ratio between 260/280 nm (Nanodrop, Thermo Scientific, USA). And all the specimens were subjected to a GAPDH gene conventional PCR assay as described previously to confirm the presence of amplifiable DNA and exclude PCR inhibition [31]. DNA was stored at −20 °C until PCR amplification.

### Nested PCR

All samples were tested at least three times for hemoplasmas (*M. ovis* and ‘*Candidatus* M. haemovis’) DNA using a nested PCR assay that amplified an approximately 500 bp fragment of the 16S rRNA gene [32]. The base sequences of four primers (A1/A2 and B1/B2) are shown in Table 1 and the binding sites of four primers are shown in Additional file 1: Figure S2. First-round PCR was performed in a total volume of 25 μl, containing 2.5 μl of 10 × LA PCR Buffer II (Mg^2+^ plus), 4.0 μl of dNTP mixture (2.5 mM each dNTP), 0.5 μl of each A1 and A2 primer (25 μM), 1.25 U of LA *Taq* polymerase (TaKaRa, Dalian, China), 16.25 μl of double distilled water, and 1.0 μl of DNA template. Cycling conditions were: 94 °C for 5 min, followed by 30 cycles of 94 °C for 30 s, 61 °C for 30 s, and 72 °C for 1 min, with a final extension at 72 °C for 7 min. Second-round nested PCR contained 2.5 μl of 10 × PCR Buffer, 2.0 μl of dNTP mixture (2.5 mM each dNTP), 0.5 μl of each B1 and B2 primer (25 μM), 0.75 U of r *Taq* polymerase (TaKaRa), 18.35 μl of double distilled water, and 1.0 μl of the first-round PCR product (diluted 10-fold). Cycling conditions were: 94 °C for 5 min, followed by 35 cycles of 94 °C for 30 s, 59 °C for 30 s, and 72 °C for 45 s, with a final extension at 72 °C for 7 min. A positive control for *M. ovis* from a naturally infected Boer goat preserved by our laboratory was used in all reactions, while double distilled water was used as a negative control. Secondary PCR products were examined by 1% agarose gel electrophoresis and visualized after GelRed (Biotium Inc., Hayward, CA) staining.

**Table 1:** Primers used for amplification of the 16S rRNA gene of hemoplasmas (*Mycoplasma ovis* and ‘*Candidatus* Mycoplasma haemovis’)

Primers	Nucleotide sequences	Target fragment/bp	Remarks
A1	5′-GGATAGCAGCCCGAAAGG-3′	1060	1st PCR
A2	5′-GCAGCCCAAGGCATAAGG-3′		
B1	5′-CTACGGGAAGCAGCAGTG-3′	506 or 489	Nested PCR [32]
B2	5′-CTCGACCTAACATCAAATACCT-3′		
16S Fw	5′-ATGCAAGTCGAACGAGTAGA-3′	1341	Conventional PCR [26]
16S Rv	5′-TGATACTTTCTTTCATAGTTTG-3′		

### PCR for 16S rRNA gene and sequencing

One hundred three positive specimens representative of different hosts, farms/flocks and geographic locations were selected and the nearly complete 16S rRNA gene sequences were amplified using the protocol described by Grazziotin et al. [26]. Primers are shown in Table 1. The 25 μl PCR mixture is the same as first-round PCR mixture of the nested PCR. PCR products were purified using Montage PCR filters (Millipore, Bedford, MA) and sequenced using a BigDye Terminator v3.1 cycle sequencing kit (Applied Biosystems, Foster City, CA) on an ABI 3730 DNA analyzer (Applied Biosystems). The nucleotide sequences were confirmed by bidirectional sequencing and by sequencing a new PCR product if necessary.

### Phylogenetic analysis

Nucleotide sequences of the nearly complete 16S rRNA gene together with reference sequences downloaded from GenBank were aligned using ClustalX 2.0 (http://www.clustal.org/) with further adjustments made manually as necessary. And then Mega 5.05 (http://www.megasoftware.net/) software was applied to conduct phylogenetic and molecular evolutionary analysis. A bootstrap phylogenetic tree demonstrating the relationship of *M. ovis* and ‘*Candidatus* M. haemovis’ genotypes to other hemoplasma species was created by the neighbor-joining method using a distance matrix corrected for nucleotide substitutions based on the Kimura 2-parameter model. A bootstrap analysis was used to assess the robustness of the clusters using 1000 replicates.

### Statistical analysis

Positive rates were compared using the chi-square test. Differences were considered statistically significant at *P* < 0.05. Analyses were performed using QuickCalcs software (GraphPad Software Inc., La Jolla, CA). The odds ratios (ORs) of the univariate analysis were calculated using measures of association along with 95% confidence intervals (CIs).

### Nucleotide sequence accession numbers

The four novel sequences determined in this study were deposited in GenBank under accession numbers: KU983740, KU983746, KU983748 and KU983749.

## Results

### Molecular prevalence of hemoplasmas (*M. ovis* and ‘*Candidatus* M. haemovis’) in sheep and goats

As shown in Fig. 1, of the 1364 blood specimens from sheep and goats, 610 (44.7%) were found to be positive for hemoplasmas (*M. ovis* and ‘*Candidatus* M. haemovis’) by PCR amplification of the 16S rRNA gene. And the positive samples distributed in 49 studied farms from seven provinces and one Aut. Reg.. In the 35 sheep and goats from Heilongjiang province, 34 (97.1%) were identified as hemoplasmas (*M. ovis* and ‘*Candidatus* M. haemovis’) -positive, while just two (5.0%) of the 40 animals in the Inner Mongolia Aut. Reg. was positive. On the other hand, the animals in three farms, including one in Henan province and two from Yunnan province were 100% positive for hemoplasmas (*M. ovis* and ‘*Candidatus* M. haemovis’) (Additional file 1: Table S1).

The infection rates of hemoplasmas (*M. ovis* and ‘*Candidatus* M. haemovis’) in sheep and goats in Heilongjiang (34/35, 97.1%) and Yunnan (246/269, 91.4%) were significantly higher than that of other provinces/Aut. Reg.. A higher prevalence was observed in grazing sheep and goats (54.4%) than household sheep and goats (33.6%). Male animals had significantly higher hemoplasmas (*M. ovis* and ‘*Candidatus* M. haemovis’) infection rates, at 60.0%, than females (41.6%). There were no significant differences between prevalence in different age groups of sheep and goats as well as that between sheep (45.8%) and goats (44.1%) (Table 2). The potential risk factors associated to percentage of infection with hemoplasmas were determined by statistical calculations. Among animal age, gender, species and feeding habits, gender (OR = 2.10, CI = 1.58–2.81) and the use of grazing (OR = 2.35, CI = 1.89–2.93) were identified as potential risk factors for hemoplasmas infection (*P* < 0.001) (Table 2).

**Table 2:** Factors associated with positive hemoplasmas (*Mycoplasma ovis and* ‘*Candidatus* Mycoplasma haemovis’) infection in sheep and goats in China

Factor	No. positive/No. examined	Infection rate (%)	OR (95% CI)	χ^2^	*p*
Age group					
< 1 year	205/450	45.6	1.05 (0.84–1.32)	0.19	0.664
≥ 1 year	405/914	44.3			
Gender					
Male	138/230	60.0	2.10 (1.58–2.81)	26.12	<0.001
Female	472/1134	41.6			
Species					
Sheep	231/504	45.8	1.07 (0.86–1.34)	0.40	0.527
Goat	379/860	44.1			
Feeding habits					
Grazing	396/728	54.4	2.35 (1.89–2.93)	59.11	<0.001
Household	214/636	33.6			

### Sequence analysis of 16S rRNA gene

Sequencing of the nearly complete 16S rRNA gene was successful for the 103 randomly selected positive specimens. Among them, analysis of the 16S rRNA gene sequences identified *M. ovis* (*n* = 56) and ‘*Candidatus* M. haemovis’ (*n* = 47). The origin of the 103 positive specimens was shown in Additional file 1: Table S2. After alignment, the obtained 103 16S rRNA gene sequences formed four sequence clusters KU983740, KU983746, KU983748 and KU983749.

KU983740 (*n* = 50) and KU983746 (*n* = 6) genotypes had 99.7% and 99.6% identity, respectively, to *M. ovis* from sheep in the USA (AF338268), and 99.9% and 99.8% identity, respectively, to a human *M. ovis* isolate form USA (KF313922). Both genotypes shared a high degree of identity (99.8%) with another human *M. ovis* isolate (GU230144), while, lower identity of them with sheep ‘*Candidatus* M. haemovis’ genotypes from Japan (AB617737) (97.3% and 97.4%, respectively) were identified. However, the other two genotypes KU983748 (*n* = 21) and KU983749 (*n* = 26) were closely related to ‘*Candidatus* M. haemovis’ (AB617737), with 99.5% and 99.4% identities, respectively, yet, their identities with *M. ovis* (AF338268) were 97.4% and 97.5%, respectively. Based on the 16S rRNA gene sequence analysis, the four novel genotypes in this study differed from *M. ovis* (AF338268) at several nucleotide positions along the full-length of the gene (Table 3).

**Table 3:** 16S rRNA gene alignment from two *M. ovis* genotypes (KU983740 and KU983746) and two ‘*Candidatus* M. haemovis’ genotypes (KU983748 and KU983749) and their comparison with two hemotropic mycoplasma sequences

Position^a^	101	118	120	125	188	201	266	272	319	340	439–455	464	466	475	507	510	570	635	648	763	1183	1274
*M. ovis* (AF338268)	G	T	G	G	G	G	T	G	C	G	AA…TG	T	G	A	C	C	G	C	G	C	C	C
KU983740(*n* = 50)	A	C	*	T	*	*	*	*	*	*	**…**	*	*	*	*	*	*	*	*	*	T	*
KU983746(*n* = 6)	A	C	*	T	*	*	*	*	*	*	**…**	*	*	*	*	*	T	*	*	*	T	*
*Candidatus* M. haemovis (AB617737)	A	C	T	T	T	A	C	A	T	A	--…--	C	T	C	T	T	T	T	A	T	T	T
KU983748(*n* = 21)	A	C	*	T	T	*	*	A	T	A	--…--	*	*	*	T	T	T	T	A	T	T	T
KU983749(*n* = 26)	A	C	*	T	T	*	*	A	T	A	--…--	*	*	*	T	T	*	T	A	T	T	T

^a^position numbers given with respect to *M. ovis* (GenBank accession number: AF338268)

*identical to the reference sequence; −, base deletion

### Phylogenetic analysis

**Fig. 2:**
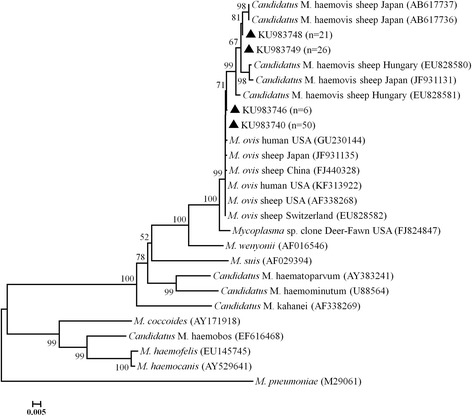
Phylogenetic relationships of *Mycoplasma ovis* and ‘*Candidatus* M. haemovis’ genotypes identified here and other hemotropic mycoplasmas. Phylogeny was inferred with a neighbor-joining analysis of the nearly complete 16S rRNA gene sequences based on distances calculated with the Kimura 2-parameter model. *Mycoplasma pneumoniae* was used as an outgroup. Bootstrap values >50% from 1000 replicates are shown on the nodes. The genotypes detected in this study are shown as *triangles*

Phylogenetic analysis revealed that the four novel genotypes accompanied by five other ‘*Candidatus* M. haemovis’ (AB617737, AB617736, EU828580, EU828581 and JF931131), six other *M. ovis* (GU230144, JF931135, FJ440328, KF313922, AF338268 and EU828582) as well as one *Mycoplasma* spp. (FJ824847) formed a separate clade. In addition, ‘*Candidatus* M. haemovis’ and *M. ovis* genotypes fell into two different groups. The two genotypes KU983740 and KU983746, representing 56 *M. ovis* sequences from the present study, were grouped in the same clade with two human genotypes from USA (GU230144 and KF313922) (Fig. 2).

## Discussion

Globally, hemotropic *Mycoplasma* spp. are emerging or re-emerging zoonotic pathogens that affect numerous animal species including humans, potentially causing loss of livestock and public health concern. *M. ovis* is prevalent worldwide, and is causative of hemolytic anemia in sheep and goats [4].

In the present study, the prevalence of hemoplasmas (*M. ovis* and ‘*Candidatus* M. haemovis’) in sheep and goats was found to be 44.7% (610/1364). A similar prevalence of *M. ovis* was previously reported in goats in Hubei, China (41.0%, 151/371) [29], while a lower rate was detected in sheep in Tunisia (6.3%, 36/573) [28]. A higher *M. ovis* prevalence was previously found in sheep in Malaysia (94.0%, 47/50) [33], Argentina (81.8%, 9/11) [9], and Hungary (51.5%, 17/33) [24]. The differences in prevalence between this study and others [9, 24, 28, 29, 33] may be caused by discrepancy in the age groups studied, as well as the number of animals, detection methods, and geographic and ecological environments. In this study, there is a striking difference in prevalence between the north-central sampling sites (Qinghai, 15.0%; Shanxi, 16.7%; Shaanxi, 7.3% and Inner Mongolia, 5.0%) versus the southern and eastern sites (Guizhou, 40.6%; Yunnan, 91.4%; Heilongjiang, 97.1% and Henan, 39.1%). The climate type of north-central sampling sites is monsoon climate of medium latitudes, whereas southern sites are subtropical monsoon climate. Invertebrate vector abundance and vegetation species in north-central sampling sites is relatively lower than southern sites. Therefore, we speculated the different climates, vegetation species and invertebrate vector abundance factors that may contribute to the apparent difference in prevalence in different provinces.

It was reported that the *M. ovis* prevalence was significantly higher in young sheep than adults (*P* < 0.001) by Rjeibi et al. [28]. However, in the present study, there was no significant difference in hemoplasmas (*M. ovis* and ‘*Candidatus* M. haemovis’) infection rates between different age groups of sheep and goats. Nevertheless, significant differences were detected among the prevalence of hemoplasmas (*M. ovis* and ‘*Candidatus* M. haemovis’) according to animal gender and feeding habits of the host animals, although no differences were observed between samples obtained from different genders by Song et al. [29]. In this study, potential risk factors were identified as animal gender and feeding habits of the host animals, providing useful information for control of the hemoplasmosis. Grazing flocks are more likely to be exposed to blood-sucking arthropods than household flocks. Household flocks received good feeding and management, which were thought to be important for the prevention from various diseases. Therefore, infections of hemoplasmas may be transmitted by blood-sucking arthropods and reusing needles during herd immunization in China.

‘*Candidatus* M. haemovis’ was first demonstrated in a sheep flock with fatal hemolytic anemia in Hungary [24], following studies reported the presence of both *M. ovis* and ‘*Candidatus* M. haemovis’ DNAs in sheep in Hungary [24], Japan [8], and the USA [27], in goats in Switzerland [25], and in *Capricornis crispus* in Japan [18]. However, there was no report documented the existence of *M. ovis* and ‘*Candidatus* M. haemovis’ in sheep and goats in China. Therefore, detection of hemoplasmas (*M. ovis* and ‘*Candidatus* M. haemovis’) by nested PCR and sequence analyses in the present study represents the first molecular evidence of the occurrence of ‘*Candidatus* M. haemovis’ in sheep and goats in China.

Phylogenetic analysis revealed that ‘*Candidatus* M. haemovis’ and *M. ovis* belong to the same clade. 16S rRNA gene homology between *M. ovis* and ‘*Candidatus* M. haemovis’ in this study was very high, at 97.3–97.5% (data not shown), revealing the close genetic relationship between them, which suggested that ‘*Candidatus* M. haemovis’ might not be a new hemoplasma species, but another copy of *M. ovis* 16S rRNA gene, which was validated by Deshuillers et al.’s report that *M. ovis* strain Michigan has two different copies of the 16S rRNA gene. The detection of multiple different 16S rRNA gene sequences in a veterinarian in Texas [22], in a severe anemia sheep in Japan [8], in goats in Switzerland [25] and in sheep in Hungary [24], might also be explained by the existence of multiple copies of the 16S rRNA gene within a single strain of *M. ovis*, each having a variable genetic composition, as reported for other bacteria [34].

In previous studies, only five hemotropic *Mycoplasma* spp., *M. haemofelis*-like [21], *M. suis*-like [35], *M. ovis* [22], ‘*Candidatus* Mycoplasma haemohominis’ [36], and ‘*Candidatus* Mycoplasma haematoparvum’ [37] organisms, were described to be infected by human beings. Sykes et al. reported the first case of human infection with hemotropic mycoplasma (*M. ovis* and ‘*Candidatus* M. haemovis’) in a veterinarian in Texas. Additionally, among the 11 hemotropic *Mycoplasma* spp. identified from 489 patients, *M. ovis*-like organism was the most prevalent [23], a species which was mainly found in small ruminants, such as deer [26, 38, 39], goats [4, 25, 33], sheep [4, 8, 24, 28, 40], and Japanese serows [18]. It is noteworthy that the nucleotide sequences of *M. ovis* (KU983740 and KU983746) in the present study had highest homology with *M. ovis* (GU230144 and KF313922) from humans (99.8% and 99.8–99.9%). The high homology of the *M. ovis* genotypes obtained in the present study with human genotypes observed here may provide additional evidence for the zoonotic potential of *M. ovis*, which were raised by Sykes et al. and then verified by Maggi et al.

## Conclusions

This study provides further evidence about the prevalence of hemoplasmas (*M. ovis* and ‘*Candidatus* M. haemovis’), and presents the first detection of ‘*Candidatus* M. haemovis’ in sheep and goats in China. Our data showed a high prevalence and widespread distribution of hemoplasmas (*M. ovis* and ‘*Candidatus* M. haemovis’) in sheep and goats. Further studies are needed to evaluate the pathogenicity of ‘*Candidatus* M. haemovis’ in sheep and goats, as well as the zoonotic potential of *M. ovis*.

## Additional file

Additional file 1: Table S1. Prevalence of hemoplasmas (*Mycoplasma ovis and* ‘*Candidatus* Mycoplasma haemovis’) of sheep and goats in different farms. **Table S2.** The origins of the 103 selected positive specimens. **Figure S1.** Collection sites (indicated by black circles) in Henan province. **Figure S2.** The binding sites of nested PCR primers. (DOCX 97 kb)

## Abbreviations

‘*Candidatus* M. haemovis’: ‘*Candidatus* Mycoplasma haemovis’
16S rRNA: 16S ribosomal RNA
Aut. Reg.: Autonomous region
*B. henselae*: *Bartonella henselae*
DNA: Deoxyribonucleic acid
EDTA: Ethylenediaminetetraacetic acid
HIV: Human immunodeficiency virus
*M. haemofelis*: *Mycoplasma haemofelis*
*M. ovis*: *Mycoplasma ovis*
*M. suis*: *Mycoplasma suis*
PCR: Polymerase chain reaction
