# Supplementary material for: Molecular characterization of hemotropic mycoplasmas (Mycoplasma ovis and ‘Candidatus Mycoplasma haemovis’) in sheep and goats in China
Source: BMC Vet Res. 2017 May 26;13:142. doi: 10.1186/s12917-017-1062-z (PMC5446696; doi:10.1186/s12917-017-1062-z)
Supplement: Additional file 1: Table S1. — Prevalence of hemoplasmas (Mycoplasma ovis and ‘Candidatus Mycoplasma haemovis’) of sheep and goats in different farms. Table S2. The origins of the 103 selected positive specimens. Figure S1. Collection sites (indicated by black circles) in Henan province. Figure S2. The binding sites of nested PCR primers. (DOCX 97 kb) [file 12917_2017_1062_MOESM1_ESM.docx]

**Additional File 1:**

**Supplementary tables and figures.**

**Table S1** **Prevalence of hemoplasmas (*Mycoplasma ovis and* ‘*Candidatus* Mycoplasma haemovis’) of sheep and goats in different farms**

| Location | | Farm (No. positive /No. examined, %) | Total (No. positive /No. examined, %) |
| --- | --- | --- | --- |
| Province/ Aut. Reg. | City/county |  |  |
| Henan | Luoyang | Farm 1(42/50, 84.0), Farm 2(8/19, 42.1), Farm 3(0/6, 0), Farm 4(0/6, 0), Farm 5 (1/19, 5.3), Farm 6(7/33, 21.2), Farm 7 (5/10, 50.0), Farm 8 (3/40, 7.5) | 66/183, 36.1 |
|  | Anyang | Farm 9(29/36, 80.6), Farm 10(0/3, 0), Farm 11 (6/7, 85.7), Farm 12 (30/30, 100) | 65/76, 85.5 |
|  | Jiaozuo | Farm 13 (9/11, 81.8) | 9/11, 81.8 |
|  | Luohe | Farm 14 (6/24, 25.0), Farm 15 (6/47, 12.8) | 12/71, 16.9 |
|  | Zhumadian | Farm 16 (2/25, 8.0), Farm 17 (1/10, 10.0) | 3/35, 8.6 |
|  | Kaifeng | Farm 18 (9/11, 81.8), Farm 19 (0/8, 0) | 9/19, 47.4 |
|  | Zhengzhou | Farm 20 (8/12, 66.7), Farm21(0/7, 0), Farm 22 (3/4, 75.0) | 11/23, 47.8 |
|  | Jiyuan | Farm 23 (11/15, 73.3) | 11/15, 73.3 |
|  | Shangqiu | Farm 24 (1/14, 7.1), Farm 25 (4/25, 16.0) | 5/39, 12.8 |
|  | Pingdingshan | Farm 26 (5/8, 62.5), Farm 27 (8/42, 19.0), Farm 28 (8/39, 20.5) | 21/89, 23.6 |
|  | Nanyang | Farm 9(0/16, 0), Farm30(2/8, 25.0), Farm 31 (1/10, 10.0) | 3/34, 8.8 |
|  | Xinxiang | Farm32(29/33, 87.9), Farm33 (11/20, 55.0) | 40/53, 75.5 |
|  | Sanmenxia | Farm 34 (1/7, 14.3) | 1/7, 14.3 |
|  |  |  | **256/655, 39.1** |
| Guizhou | Guiyang | Farm35 (15/17, 88.2), Farm 36 (8/42, 19.0) | 23/59, 39.0 |
|  | Qinglong | Farm 37 (1/19, 5.3) | 1/19, 5.3 |
|  | Longli | Farm 38 (8/16, 50.0) | 8/16, 50.0 |
|  | Panxian | Farm 39 (20/34, 58.8) | 20/34, 58.8 |
|  |  |  | **52/128, 40.6** |
| Inner Mongolia | Sonid Right Banner | Farm 40 (2/20, 10.0) | 2/20, 10.0 |
|  | Shangdu | Farm 41 (0/20, 0) | 0/20, 0 |
|  |  |  | **2/40, 5.0** |
| Shanxi | Wanrong | Farm 42 (2/12, 16.7) | **2/12, 16.7** |
| Shaanxi | Linyou | Farm 43 (2/14, 14.3), Farm 44 (4/18, 22.2) | 6/32, 18.8 |
|  | Jingyang | Farm 45 (1/28, 3.6) | 1/28, 3.6 |
|  | Gaoling | Farm 46 (2/35, 5.7) | 2/35, 5.7 |
|  | Fuping | Farm 47 (6/110, 5.5) | 6/110, 5.5 |
|  |  |  | **15/205, 7.3** |
| Yunnan | Kunming | Farm 48 (6/13, 46.2), | 6/13, 46.2 |
|  | Xundian | Farm 49 (40/51, 78.4) | 40/51, 78.4 |
|  | Longling | Farm50(78/80,97.5), Farm51 (54/55, 98.2), Farm52 (20/20, 100), Farm 53 (8/10, 80.0), Farm 54 (40/40, 100) | 200/205, 97.6 |
|  |  |  | **246/269, 91.4** |
| Qinghai | Xining | Farm 55 (3/20, 15.0) | **3/20, 15.0** |
| Heilongjiang | Qiqihar | Farm 56 (34/35, 97.1) | **34/35, 97.1** |
| **Total** |  |  | **610/1364, 44.7** |

**Table S2 The origins of the 103 selected positive specimens**

| Hemoplasmas | Isolates | Source of 103 sequences | | |
| --- | --- | --- | --- | --- |
|  |  | Province/ Aut. Reg. | City/county | Farm (No. selected) |
| *M. ovis* | KU983740 (n=50) | Henan | Luoyang | Farm 1 (2), Farm 2 (1), Farm 5 (1), Farm 6 (2) |
|  |  |  | Anyang | Farm 9 (2) |
|  |  |  | Luohe | Farm 14 (1), Farm 15 (1) |
|  |  |  | Zhengzhou | Farm 20 (1), Farm 22 (1) |
|  |  |  | Pingdingshan | Farm 26 (1), Farm 27 (1), Farm 28 (1) |
|  |  |  | Xinxiang | Farm 32 (2) |
|  |  | Guizhou | Guiyang | Farm 35 (2) |
|  |  | Inner Mongolia | Sonid Right Banner | Farm 40 (2) |
|  |  | Shanxi | Wanrong | Farm 42 (2) |
|  |  | Shaanxi | Linyou | Farm 43 (2), Farm 44 (2) |
|  |  |  | Jingyang | Farm 45 (1) |
|  |  |  | Gaoling | Farm 46 (2) |
|  |  |  | Fuping | Farm 47 (3) |
|  |  | Yunnan | Kunming | Farm 48 (4), |
|  |  |  | Xundian | Farm 49 (4) |
|  |  |  | Longling | Farm 50 (6), |
|  |  | Qinghai | Xining | Farm 55 (3) |
| *M. ovis* | KU983746 (n=6) | Henan | Zhumadian | Farm 16 (1), Farm 17 (1) |
|  |  | Heilongjiang | Qiqihar | Farm 56 (4) |
| ‘*Candidatus* M. haemovis’ | KU983748 (n=21) | Henan | Anyang | Farm 11 (2) |
|  |  |  | Kaifeng | Farm 18 (2) |
|  |  |  | Jiaozuo | Farm 13 (2) |
|  |  |  | Jiyuan | Farm 23 (3) |
|  |  |  | Shangqiu | Farm 24 (1), Farm 25 (2) |
|  |  |  | Nanyang | Farm 30 (2), Farm 31 (1) |
|  |  |  | Sanmenxia | Farm 34 (1) |
|  |  | Heilongjiang | Qiqihar | Farm 56 (2) |
|  |  | Guizhou | Guiyang | Farm 36 (2) |
|  |  |  | Qinglong | Farm 37 (1) |
| ‘*Candidatus* M. haemovis’ | KU983749 (n=26) | Henan | Luoyang | Farm 7 (2), Farm 8 (1) |
|  |  |  | Anyang | Farm 12 (2) |
|  |  |  | Xinxiang | Farm 33 (2) |
|  |  | Guizhou | Longli | Farm 38 (2) |
|  |  |  | Panxian | Farm 39 (3) |
|  |  | Yunnan | Longling | Farm 51 (4), Farm 52 (4), Farm 53 (2), Farm 54 (4) |

**Figure S1 Collection sites (indicated by black circles) in Henan province.**


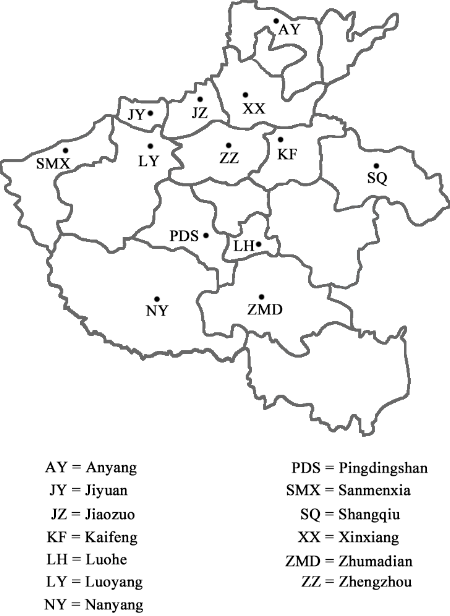


**Figure S2 The binding sites of nested PCR primers.** The four primers were designed based on the homologous regions of 16S RNA gene sequence of *M. ovis* (AF338268) and *Candidatus* M. haemovis (AB617737). *Candidatus* M. haemovis (AB617737) missed the corresponding 17 bp fragment ranging from 439bp to 455 bp in *M. ovis* (AF338268).
